# The 3Fs as conditions of being: relational ontology and equine welfare in disaster response and reconciliation

**DOI:** 10.3389/fvets.2026.1922532

**Published:** 2026-08-18

**Authors:** Dana Grinde

**Affiliations:** School of Health in Social Science, University of Edinburgh, Edinburgh, United Kingdom

**Keywords:** 3F framework, autoethnography, equine flourishing, multispecies justice, onto-epistemology

## Abstract

Disaster relief work requires the collaboration of many stakeholders and can be viewed through a One Health lens, emphasising transdisciplinarity and an openness to different ways of knowing as well as an appreciation of the interdependence of human, animal, environmental health, and welfare. This perspective piece provides an autoethnographic account of a wildfire response in Western Canada in which a herd of free-ranging horses was rescued outside of conventional disaster management protocol. Through lived experience, the convergence between equine welfare, reconciliation, and relational ontology became visible. This has implications both for disaster management and for Fraser’s 3Fs of equine welfare, offering a philosophical expansion that recognises conditions of being that, together, co-constitute the potential for flourishing. The perspective advanced here aims to shift equine welfare practice from the management of discrete variables toward a practice that is defined by relational integrity and accountability, in which humans and horses are bounded beings, where flourishing results from honouring the dynamic and entangled system—understood as a shared ground of being—that humans, animals, and land belong to. This work contributes to One Health approaches by foregrounding and exemplifying relational knowledge, which is often excluded from traditionally positivist disciplines. This work situates equine welfare within broader ethical movements, including decolonial projects, efforts toward multispecies justice, and the development of One Health approaches. This alignment extends responsibility beyond individual caregivers and invites a reorientation of welfare science toward a relational ontology, offering a framework that is both practically grounded and philosophically expansive.

## 1 Introduction

In the face of disasters such as floods and wildfires, the enormity and immediacy of the crisis tests our preparedness and our understanding of what is important and should be prioritised. It also brings into focus our relationships, our responsibilities (1–3) and our very understanding of our relational lives. There is an increasing sense that disaster relief operations need to recognise how entangled human health is with that of the more-than-human world. This has led to calls for One Health to sit at the centre of emergency and resilience planning (4). The One Health principles (5) emphasise transdisciplinarity, both modern and traditional forms of knowledge, and a broad representative array of perspectives; when applied to disaster relief this raises questions about how contemporary western assumptions about reality, with its emphasis on analysis and architectivity can learn from indigenous science with its emphasis on relationality and connectivity (6, pp. 833–835). Against this backdrop, the present perspective piece responds to the aims of this special issue by bringing a relational and interdisciplinary lens to equine welfare. At a time when veterinary medicine is increasingly engaging with diverse epistemologies and philosophical perspectives, re-examining established welfare frameworks through a relational lens opens an opportunity to expand, not replace, existing approaches to understanding equine wellbeing.

Fraser’s 3F framework (7–9) offers measurable indicators of equine wellbeing and, importantly, centres the horse’s perspective. While this approach has contributed to improving welfare standards [e.g., (10–12)], it remains grounded in a model that treats welfare as the assessment and management of discrete variables. In so doing, it risks reducing complex lived experiences to detached proxies (13), analysed on a species-by-species basis and ignoring the importance of the horse’s experience in her own right (14). This perspective piece proposes that this model of welfare, despite acknowledging the interrelation of health factors, decontextualises and fragments what is an irreducibly relational and entangled system.

This work draws on an autoethnographic account of a wildfire disaster response involving the rescue of a herd of free-ranging horses in Western Canada to inform and propose a conceptual shift: from understanding the 3Fs as equine welfare indicators to understanding them as mutual conditions of being. This perspective situates equine welfare within a relational ontology, where horses, humans, and environments are not separate entities interacting, but co-constituted participants within a shared ecological and ethical field.

The autoethnographic method applied in this article helps to articulate how the 3Fs can be understood as conditions of being. It is through the lived experience of my own alignment with an expanded 3Fs framework—the recognition of conditions for flourishing as they emerged across multiple practices (disaster management, reconciliation, and equine welfare)—that this insight is generated. This alignment resonates with Barad’s concept of ‘intra-action’, a perspective that frames welfare as something emerging through dynamic and entangled relations among horses, humans, and land rather than a fixed property located within individual animals. Similarly, Haraway’s notion of ‘becoming-with’ and Cousquer et al.’s (15) usage of “intra-dependence”, in relation to veterinary teaching, points to the ways multispecies beings are constituted through shared practices of care, vulnerability, and response. By putting these theorists into conversation with Fraser’s 3Fs, it is apparent that disparate disciplines are converging toward a relational ontology. This ontological convergence from different theoretical starting points suggests that this specific horse rescue is not an isolated, idiosyncratic anecdote, but a localised manifestation of a much broader relational reality. It is from this perspective that this autoethnographic account advocates for relational accountability and a multispecies care ethics that expands and deepens contemporary understandings of equine welfare. This paper first situates equine welfare within dominant positivist paradigms before turning to relational ontology and Indigenous scholarship to reconceptualise Fraser‘s 3F framework through lived multispecies relational practice.

## 2 Extending Fraser’s 3Fs

Traditional approaches in veterinary welfare science are predominantly grounded in positivist methodologies that aim to isolate measurable variables such as nutrition, mobility, and environmental complexity (16–19). While these approaches have substantially advanced practical welfare assessment, they are unable to capture the relational and systemic nature of life (20, 21); under-theorising research and overlooking important onto-epistemological assumptions in veterinary science (22).

Empirical welfare models regularly rely on reductionist approaches that separate phenomena into discrete parts; this method struggles with multi-layered social and behavioural phenomena because its research design favours simplicity (for the sake of management), over the messiness of reality (23, 24). As a result, welfare risks being conceptualised as a set of compliance-based benchmarks rather than as a dynamic condition emerging from relational participation in a shared world. This limitation calls for a conceptual expansion that can account for the entangled nature of lived experience.

Fraser’s 3F framework begins to move us in the direction of a relational conceptualization of wellbeing where a horse’s environment, social life, and freedom are both inter-connected and directly connected to physiological and behavioural outcomes. Fraser states:

I hope to introduce the idea that these three needs are actually hard to isolate individually, and could instead be looked at as one very important, intertwined need of the horse. Each need—friends, forage, and freedom—is like a cog, turning the wheels of a machine. Take one away, and the machine will not operate as intended. The same could be said for the horse; horses need friends, forage, and freedom to operate and live happily and healthily. Removing one will have a negative effect on how the whole horse operates (9), para. 1.

Fraser makes an important conceptual move by recognising that equine wellbeing does not emerge from isolated variables, but through entangled relational conditions, whilst still committing the error that has blighted modernity by drawing on a machine metaphor. By recognising friends, forage, and freedom as mutually constitutive rather than as separable needs, the 3F framework begins to challenge the atomistic logic that often underpins conventional welfare science. The horse is no longer understood as an individual organism responding mechanically to environmental inputs; she becomes a being who’s flourishing emerges through participation in a dynamic relational system. To realise the paradigm shift that allows us to break free from the fragmented logic of western science (6), the hidden assumptions rooted in modernity that are reflected in Fraser’s choice of metaphor (cogs and machines) and verb (operate) need to be held up to scrutiny and replaced.

The autoethnographic reflection that follows aims to extend Fraser’s framework further, asking: if these conditions function as an inseparable system within the horse’s world, then the boundaries of that system cannot end with the horse alone. Human caregivers, social practices, built environments, and cultural orientations toward animals also participate in the system that shapes the conditions from which wellbeing emerges (23).

## 3 Autoethnographic insight

In 2017, I was working for the Canadian Red Cross on their National virtual disaster management team. We processed 1,000s of escalations for the wildfire response in British Columbia. Our primary directive was to help victims of the fires meet their basic needs. One day, my supervisor called me over to her desk, “I have an interesting file for you” she said. A herd of horses had been spotted down a remote logging road. They were starving, far from food and water, and a long way from their natural range and ancestral caretakers. What made the file interesting is that the horses did not belong to anyone, they were wild, and while we did assist with the care of animals, it was typically when they were classified as pets or livestock. These horses were neither assets nor dependents to humans; under our framework they were a sad loss, a part of the burnt landscape.

I remember the heaviness of that moment. I was fearful that nothing could be done all the while feeling the intense call to respond to their suffering. I felt a strong tension between what the moment was asking of us and our standard mode of operation. Our desperation led us to an important but often overlooked feature of our disaster framework: question number eight in the directive allowed for discretion and advocacy in ‘exceptional circumstances’. Though the written policy prioritized human-centric metrics, the spirit of the directive left space for caseworker judgement when the standard protocol did not fit the ethical imperative.

My supervisor was not from this region and was not familiar with this specific herd of horses. She did, however, have a lens that was different from my own. She was attuned to a deeper relational reality and was aware of a stewardship ethics that was not familiar to me. Her approach to the crisis was not defined in the same way as the logistical efficiency of the disaster management world; her worldview was grounded in relational responsibility. As she later explained to me, for both her own Indigenous community and the Indigenous communities of the Interior plateau, these horses were kin. For generations, these herds had lived alongside Indigenous communities and were stewarded through a reciprocal relationship of care and coexistence that predated colonial land management frameworks. The horses were not separate entities that needed management; they were relatives whose suffering was linked in inextricable ways to the land and people. If it had not been for my supervisor’s appreciation and understanding of their importance, we would not have been able to participate in their rescue.

What added complexity to the response, was that a majority of the land and people who were affected were Indigenous. We had training as caseworkers, in Reconciliation and Indigenous Studies. Each of our outreach teams had an Indigenous Relations Officer, and rather than imposing aid on communities, our protocol was to wait for an invitation, and we consulted with and followed the Chief of each Nation’s requests for how aid was to be delivered. The conceptual approach of our policy was to build a framework that paralleled and respected Indigenous paradigms. Elders and other band members were consulted, and efforts were made to work alongside each other courteously, without convergence or appropriation. What I experienced in the rescue operation had a different felt sense. It did not feel like parallel efforts toward the same end; it felt like belonging to a shared web that had been activated.

The best word to describe what unfolded is rhizomatic (25, 26). As opposed to standard procedure, the response was non-hierarchical, open ended, and emphasized a horizontal, messy, network of connections. Efforts branched and multiplied, were multidirectional and not clearly defined. There was also a feeling of surrender and trust. Normally, a caseworker takes notes and documents everything. This was more like a game of telephone. Once I made the initial phone calls, it was out of my hands and out of my control. I served as a touch point between people. The horses’ needs were met by people who had similarly been activated through the network, people who knew them and who had knowledge of how to best care for them. They were brought to safety, they were fed, and they were led to water. We did not proactively apply the 3Fs as a welfare checklist to help them survive; we connected into a network of care that understood what horses needed through generations of caring alongside them. Their welfare was a consequence of the right relation (27) between caseworker, the people on the ground, the land, and the horses.

This experience, the situational ethical responsiveness and the emergent relational understanding, felt like what Wilson (28) describes in “Research is Ceremony”, as relationality. It felt like an active and sacred process, where knowledge, identity, and reality itself emerged directly through our connection. Reconciliation here did not feel like what I learned in the Indigenous Canada course. It was something lived out, and it became visible to me through moments of coordinated care and common purpose. This connection between animals, people, and land enacted reconciliation as a sort of ground. Not reconciliation as policy or the inclusion of Indigenous content in a parallel framework, but as a lived and shared reality, one we stumbled into because the horses suffering called us into relationship, into action.

The participatory knowledge that I gained in this experience brought to mind this idea that the 3F framework for equine welfare and the conditions that supported the effective response to the horses’ suffering were not just parallel; they were one in the same. This story begins to make clear that the horses and the people trying to rescue them exist within the same relational weaving, and the same conditions, freedom, friendship, and forage, that govern the flourishing of horses, also governs the conditions of a broader wellbeing for all.

## 4 Relational ontology and the 3Fs

I first shared this story at the European Congress of Qualitative Inquiry, 2026. My talk highlighted the fact that when there’s space in policy, much like a crack in the pavement, life can unfold in a way that is aligned with something more like what really is, I called this: ontological alignment, asking what is revealed when something is neither forbidden nor determined? I believe this question mirrors what Fraser was pointing to in her series “The Horse’s 3Fs: Friends, Forage, and Freedom” (7–9). She explores the potential of who a horse might become when they are free to be as they are.

It is necessary to point out here, that across generations, and long before the emergence of western scientific frameworks (rooted in analytical philosophy), Indigenous people in what is now called Canada have engaged with “sentient environments [and] with cosmologies that enmesh people into complex relationships between themselves and all relations” (29, p. 6). Though there has been an ontological turn more recently in western scholarship, with an increasing acknowledgment of the sentience and agency of land, animals, and people (29), these orientations are neither novel nor new. Relational ontologies have been articulated, lived, and theorised within Indigenous philosophies for a millennia (29, p. 6). Watts (30) argues that Indigenous scholarship has strongly influenced many strands of post-humanism and the ontological turn itself, even though this influence remains largely unacknowledged. The new One Health definition recognises the need for traditional forms of knowledge to be integrated into transdisciplinary multisectoral collaboration (5); this will challenge western science to address medical colonisation and decolonise its practices. In the case of the emergency response I’ve recounted here, there is hesitation in wanting to acknowledge relational ontology for fear of distorting, subsuming, or homogenising the particularities of Indigenous thought. The risk is that recognition becomes a form of erasure, where these longstanding knowledge systems are retroactively folded into emerging western frameworks. The parallel framework the Canadian Red Cross was trying to articulate, was an effort to avoid this erasure.

What is felt in this narrative though, is that this convergence was not one of equivalence, where distinct knowledge systems collapsed into sameness; it was one of resonance, where we enacted a shared metaphysical orientation, found in lived practice rather than an abstractly defined theory (31). In this way, the alignment was not conceptual; it was enacted and emergent, and it arose through integrity and care, through attention and participation in a relational world. From this perspective, engagement across difference wasn’t simply the inclusion or appropriation of another worldview, but something closer to ontological alignment. Indigenous scholars have long articulated relational and living worlds and so the question then becomes, what might it mean to finally meet on relational ground, recognising that it is a way of being that has and is already being lived?

Considering the 3Fs as conditions of being within a relational ontology, invites us to look at the concepts of friendship, forage, and freedom beyond the horse. Kimmerer (32) helps us shift our understanding of friendship, where friendship is less a matter of choosing companions than a practice of mutual care, gratitude, and responsibility toward all beings. In her own work on disasters and environmental crises, Celermajer et al. (33) highlight how both humans and non-humans become bound together through shared vulnerability, these communities of fate emerge because beings are exposed to the same conditions of suffering and survival alongside one another. This reframes Fraser’s assertion that ‘horses need other horses’ by expanding the circle of belonging, where horses are kin to all beings, embedded in a community of life that transcends species boundaries. My autoethnographic reflection enacted this; the horses’ suffering called beyond their immediate companions, pulling farther reaches of the web to which they belong, activating caseworkers a province away. In this same vein, belonging can be expanded beyond social relationships to include land and place. Fraser’s concept of forage can move toward this broader understanding as well. She writes, “When an animal’s physical needs aren’t met, their emotional needs also suffer; mind and body are linked, and horses who are denied constant access to forage can exhibit many behaviour problems” (8), para. 9. While Fraser emphasises the physiological and psychological importance of forage, her concept also draws attention to a horse’s embeddedness within the landscapes that sustain them. Forage is not only about food; it is relationship with place. Both Kimmerer and Watts extend this understanding of belonging by describing land as a living relation rather than as a passive resource. Watts (30) argues that land is not separate from us but constitutive of who we are, and Kimmerer (32) reminds us that the places that sustain us are also members of our communities. The wellbeing of horses and humans alike depends upon the participation in relationship with the lands that nourish them. Because we inhabit shared places and depend on and exist within shared ecologies, our ground of being is mutual.

Given this expanded understanding of friendship and forage then, it follows that freedom becomes the capacity of a being to participate in the relationships, communities, and environments through which they become themselves. Thought of in this way, freedom illuminates the common ground between horse welfare and reconciliation. Both are concerned with restoring the conditions under which beings can meaningfully participate in the worlds that sustain them, making flourishing possible. The space in the disaster-response policy for caseworker discretion afforded me the freedom to respond to the suffering of the horses that called me into action because of the relational field to which I belong.

## 5 Discussion

Matthew Calarco’s classic, *Zoographies* (34), demonstrates the limitations of the animal-human binary and highlights how continental philosophies have excluded non-human beings from ethical consideration. While post-species and post-human lenses expand the circle of moral concern, relational ontology pushes this project further. Rather than adding beings to the list of ethical consideration, it challenges the assumption that beings exist independently of one another, prior to relationship. Within this perspective the horse is not an individual who acquires friends, forage, and freedom. Instead, these are social, physical, and agential conditions through which her being becomes possible.

This moment in time calls for “more than innovation and risk management, it calls for a radical shift in how we attend, sense, and act: […] capacities that are at the very core of responding to the ruptures and challenges of our time” (35). The autoethnographic account presented here is one situated glimpse into that shift, illustrating how lived experience can reveal relational realities that are easily obscured by more detached forms of inquiry.

If horses, humans, and environments are not separate entities that subsequently interact, but are instead co-constituted through their relationships, then the ways we come to know about them must also take this into account. Recognising that the boundaries of being are porous dissolves the subject/object divide and invites knowledge through participation and connection rather than detached observation. Haraway (36) describes this as “situated knowledge”, recognising that all knowledge is partial and emerges from particular relationships and contexts. If reality is encountered through participation, then participatory methods and lived experience become philosophically appropriate, an epistemic resource as opposed to methodological weakness.

Importantly, the convergence explored here should not be mistaken for discovery. As noted, Indigenous scholarship and cosmologies have long inhabited and described relational ontologies that are grounded in reciprocal relationships between humans, animals, and land. This perspective affords us an opportunity for respectful dialogue and engagement with Indigenous knowledge traditions whose relational understanding long predates contemporary relational philosophies. Coming to shared ground calls for humility and reorients reconciliation toward a practice that is enacted and lived.

Another consequence of this shift is a deepening of multispecies justice. Through this lens we might ask whether the relational conditions that enable humans, animals, and ecosystems to flourish are being sustained instead of looking only to whether individual animals are treated fairly. Justice in this way, like welfare, becomes a property of healthy relationships.

As animals and humans collide with increasing intensity and frequency through globalization, natural and man-made disasters, and through reconciliatory efforts, the clear boundaries between species and land dissolve, complicating our interactions and calling for knowledge, collaboration, and frameworks that transcend disciplines, as outlined in One Health’s new definition for a sustainable and healthy future (5). Pollock writes that despite the reality of human-animal-land entanglement, veterinarians remain an underrepresented voice on disaster management committees and within standard emergency management frameworks (4). Yet, “veterinary professionals bring more than clinical care: they support human decision making, community wellbeing and economic recovery; they understand disease control, biosecurity and the human-animal bond” (4), para. 5. Veterinary expertise is not diminished or blurred through this lens; in fact, a relational perspective expands the contexts in which that expertise becomes essential, recognising veterinarians as necessary contributors to the transdisciplinary responses that are called for more and more by contemporary crises.

The ontological shift explored through this autoethnographic account brings equine welfare practices into harmony with One Health (5), global decolonial efforts [e.g., (29, 37)], and efforts toward multispecies justice [e.g., (38–40)]. As Roy (31) suggests, this metaphysical orientation cultivates a conceptual and experiential capacity that allows us to participate more effectively in reality’s ongoing becoming. In so doing, it provides us with the concepts and language through which veterinary science and medicine, disaster management, Indigenous knowledge systems, environmental sciences, and multispecies ethics can engage one another while retaining their distinct contributions. The outcome is a richer understanding of how diverse forms of knowledge can work together on shared ground to cultivate the conditions under which humans, animals, and ecosystems flourish.

## Data Availability

The original contributions presented in the study are included in the article/supplementary material, further inquiries can be directed to the corresponding author.
